# Effect of aripiprazole once-monthly 400mg (AOM400) on hospitalisation prevention and use of healthcare resources in schizophrenia patients, a study based on real clinical practice on schizophrenia: AMBITION Study

**DOI:** 10.1192/j.eurpsy.2023.1052

**Published:** 2023-07-19

**Authors:** V. Sanchez-Gistau, M. J. Moreno, S. Gómez-Lus, A. Sicras Mainar, B. Crespo-Facorro

**Affiliations:** 1 Early Intervention Psychosis Service, Hospital Universitari Institut Pere Mata, IISPV, Universitat Rovira i Virgili, CIBERSAM, Reus; 2 Medical department, Otsuka-Lundbeck Alliance, Barcelona; 3 Atrys Health, S.A., Madrid; 4 Department of Psychiatry, School of Medicine, University Hospital Virgen del Rocio, CSIC, IBiS-CIBERSAM, University of Sevilla, Sevilla, Spain

## Abstract

**Introduction:**

Schizophrenia is a large and increasing burden for patients from early stages of the disease. Long-acting injectable antipsychotics (LAIs), like aripiprazole once-monthly 400 mg (AOM400), have demonstrated an improvement in treatment adherence compared to oral formulations, with a consequent reduction in time to remission and risk of relapse.

**Objectives:**

This study aims to compare the hospitalisation rate in individuals with schizophrenia who started their treatment with AOM400 or atypical oral antipsychotics (OA) in a real-world setting in Spain.

**Methods:**

This is an observational and retrospective study based on the electronic medical records of the BIG-PAC database. Adults diagnosed with schizophrenia who initiated treatment with AOM400 or atypical OA (olanzapine, risperidone, paliperidone, aripiprazole or asenapine) from 01/01/2017 to 31/12/2019 were included. A 1:1 propensity score matching (PSM) was conducted to match individuals from both cohorts. Healthcare resource use and treatment persistence (with AOM400 or OA) were also analysed after 12 months.

**Results:**

After the PSM, 1,017 individuals with similar baseline characteristics were included in each cohort (total population: 2,024 individuals). At index date (treatment initiation) patients were 41.4 years (standard deviation, SD: 10.6), 54.6% were male and had received 1.6 (SD: 0.9) previous antipsychotic treatments. During the follow-up period, the AOM400 cohort had a 40% lower risk of hospitalisation than the OA cohort (hazard ratio, HR: 0.60 [95% confidence interval, CI: 0.49 – 0.74]). The median time to the first hospitalisation was longer in individuals with AOM400 compared to those with OA (197 compared to 174 days; p<0.004), whereas median length of hospital stay were shorter (6 and 11 days for AOM400 and OA, respectively; p<0.001). The AOM400 cohort also required fewer visits to primary care, specialized care and emergency rooms than the OA cohort (p≤0.005). After 12 months, the AOM cohort was more persistent than the OA cohort (64.9% compared to 53.7%; p<0.001).

**Conclusions:**

AOM400 reduces the number and duration of hospitalisations and improves treatment persistence compared to atypical OA. Our results suggest that the use of AOM400 may reduce the burden of schizophrenia in Spain.

**Disclosure of Interest:**

None Declared

